# LINE-1 methylation status and survival outcomes in colorectal cancer patients: A systematic review and meta-analysis

**DOI:** 10.1016/j.heliyon.2025.e42410

**Published:** 2025-01-31

**Authors:** Akbar Bagheri, Tohid Jafari-Koshki, Leila Alizadeh, Mortaza Raeisi, Yaghoub Moaddab, Abbas Karimi

**Affiliations:** a Department of Molecular Medicine, Faculty of Medical Sciences, Tabriz University of Medical Sciences, Tabriz, Iran; b Molecular Medicine Research Center (MMRC), Department of Statistics and Epidemiology, Faculty of Health, Tabriz University of Medical Sciences, Tabriz, Iran; c Gastroenterology and Liver Diseases Research Center, Tabriz University of Medical Sciences, Tabriz, Iran; d Hematology and Oncology Research Center, Tabriz University of Medical Sciences, Tabriz, Iran

**Keywords:** Colorectal cancer, Long interspersed nuclear element-1, Methylation, Survival

## Abstract

Colorectal cancer (CRC) is a leading cause of cancer-related mortality worldwide. DNA hypomethylation, particularly of Long Interspersed Nuclear Element-1 (LINE-1) repetitive sequences, has been implicated in CRC development and progression. The purpose of this meta-analysis and systematic review was to evaluate the prognostic value of LINE-1 methylation level in patients with CRC. Relevant studies were identified through systematic database searches, and summary hazard ratios (HRs) with 95 % confidence intervals (CIs) were calculated for relations between LINE-1 hypomethylation and overall survival (OS), disease-free survival (DFS), and cancer-specific survival (CSS). The current systematic review protocol was registered on International Prospective Register of Systematic Reviews (PROSPERO, CRD42024496615). A total of 19 studies comprising 8169 CRC patients were included. The pooled analysis showed no significant association between LINE-1 hypomethylation and OS in the overall population (HR = 1.70, 95 % CI: 0.97–2.96). However, in stage II/III patients, LINE-1 hypomethylation was significantly associated with poorer OS (HR = 1.92, 95 % CI: 1.26–2.91) and DFS (HR = 2.81, 95 % CI: 1.33–5.93). No significant association was found between LINE-1 hypomethylation and CSS (HR = 1.39, 95 % CI: 0.68–2.83). Substantial heterogeneity was observed across studies. The study suggests that LINE-1 methylation level could be a valuable prognostic biomarker for advanced-stage CRC patients, potentially improving personalized care and highlighting areas for future research to establish standardized methodologies.

## 1 Introduction

Colorectal cancer (CRC) is the third most common cancer and the second leading cause of cancer death worldwide. As stated by Globocan 2020, CRC, with 1.9 million new cases and 935,000 cancer-related deaths, sequentially corresponding to 9.4 % (5.8 % for colon and 3.4 % for rectum tumors) and 10.0 % (6.0 % for colon and 3.8 % for rectum) of deaths and all new cancer cases, attracts attention as a global health concern [1]. CRC is a complex disease, with multiple factors contributing to the development of CRC. In countries with medium and high Human Development Index (HDI) scores, where there is a trend towards adopting a 'Western' lifestyle, both the incidence and mortality of CRC have significantly increased [2].

This cancer is a multi-pathway disease that can be classified into four subtypes based on genomic and epigenomic instabilities: chromosome instability (CIN), microsatellite instability (MSI), CpG island methylator phenotype (CIMP), and DNA global hypomethylation. According to the tumor-node-metastasis (TNM) staging method, CRC patients are categorized from stage I (the least advanced stage of the disease) to stage IV (the most advanced stage) [3,4]. The 5-year survival rate for CRC patients varies by stage: approximately 64 % for localized CRC and 12 % for metastatic CRC [5].

Genetic and epigenetic factors significantly influence the initiation and progression of CRC, with DNA global hypomethylation being a common feature. The prognostic significance of these pathways, especially the methylation status of LINE-1 elements, which comprise approximately 18 % of the human genome, is the subject of most investigation [6,7]. The activation of LINE-1 through hypomethylation can disrupt genomic stability and may be implicated in the development of most cancers [8,9]. LINE-1 consists of two main open reading frames (ORFs) that encode the proteins involved in endonuclease and reverse transcriptase activities [10]. CpG dinucleotides at the LINE-1 promoter are methylated in normal cells, leading to LINE-1 repression and transcription silencing. LINE-1 promoter hypomethylation improves the availability of RNA polymerase II and the other regulatory factors to start or regulate the transcription of these elements [11].

Several studies have investigated the LINE-1 methylation level and clinical outcome of CRC progression. These studies have demonstrated that a low LINE-1 methylation level was closely associated with a shorter recurrence-free survival (RFS) time [12] and shorter OS time [13]. Furthermore, patients with low LINE-1 methylation status have been identified to have lower five-year DFS and OS compared to those of patients with normal LINE-1 methylation [9]. Published data demonstrated that as LINE-1 methylation decreased, the 5-year CSS probability in CRC patients decreased [14].

In accordance with the DATECAN initiative's standardized definitions for time-to-event (TTE) end-points in CRC as described in 2020 [15], our study endeavors to integrate and examine the relationship between these end-points and LINE-1 methylation level in CRC patients. Our goal is to meticulously analyze LINE-1 methylation levels and their prognostic significance, in line with the established consensus definitions: OS as death from any cause; DFS encompassing all causes of death, second primary colon cancers, and relapses; and CSS which includes deaths attributable to recurrence, treatment-related mortality, and deaths from colon cancer. By aligning our research with these standardized end-points, we aim to provide valuable insights into the prognostic landscape of CRC, which may guide future clinical practices. Our study also seeks to build upon and refine previous findings, addressing limitations such as the absence of stage-specific HR comparisons and highlighting standardized cut-off for LINE-1 methylation. Through the establishment of a clear hypothesis and research questions, we aspire to deliver a robust analysis of the role of LINE-1 methylation in CRC prognosis, potentially impacting future clinical approaches.

## 2 Methods

### 2.1 Study registration

This systematic review and meta-analysis was conducted in accordance with the Preferred Reporting Items for Systematic Reviews and Meta-Analyses (PRISMA) guidelines and is registered with PROSPERO (CRD42024496615).

### 2.2 Literature search and selection criteria

According to the PRISMA2020 guidelines for identifying eligible studies a comprehensive literature search by two of the authors (AB and AK) was conducted across PubMed, Science Direct, Cochrane Library, and the Registry of Controlled Trials up to February 12, 2023. The selected search terms were (“Colorectal Neoplasm” OR “Neoplasm, Colorectal” OR “Neoplasms, Colorectal” OR “Colorectal Tumors” OR “Colorectal Tumor” OR “Tumor, Colorectal” OR “Tumors, Colorectal” OR “Colorectal Cancer” OR “Cancer, Colorectal” OR “Cancers, Colorectal” OR “Colorectal Cancers” OR “Colorectal Carcinoma” OR “Carcinoma, Colorectal” OR “Carcinomas, Colorectal” OR “Colorectal Carcinomas”) AND (“LINE Repeat Sequences” OR “LINE Repeat Sequence” OR “Repeat Sequence, LINE” OR “Repeat Sequences, LINE” OR “Sequence, LINE Repeat” OR “Sequences, LINE Repeat” OR “Long Interspersed DNA Sequence Elements” OR “LINE-1 Elements” OR “Element, LINE-1” OR “Elements, LINE-1” OR “LINE 1 Elements” OR “LINE-1 Element” OR “L1 Elements” OR “Element, L1” OR “Elements, L1” OR “L1 Element” OR “Jockey Elements” OR “Element, Jockey” OR “Elements, Jockey” OR “Jockey Element”). In addition to these terms, our search strategy included “Survival Analysis”, “Overall Survival”, “Disease-Free Survival”, “Progression-Free Survival”, “Time to Recurrence”, “Cancer-Specific Survival”, “Kaplan-Meier Estimate”, “Prognosis OR Prognoses”, “Outcome OR Outcomes”, “Prognostic Value”, “Prognostic Factor”, “Prognostic Indicator”, “Recurrence Risk”, “Predictive for Outcome”, and “Recurrence”.

To complement the articles identified through the systematic search we did not apply any restrictions in terms of language (languages), type of study (research types), and publication date, also manually studied the citation lists of included studies and previous systematic reviews. The entire search strategy and terms used are provided in Supplementary Table 1, while a summary of the search results across different databases is presented in Supplementary Table 2.

Studies were chosen based on the following criteria: (1) patients with stage I–IV CRC; (2) randomized clinical trials or prospective/retrospective cohort studies; (3) studies with analyzed LINE-1 methylation level; (4) studies reporting or providing sufficient information about at least one of patient outcomes data including OS, CSS, DFS, RFS; (5) article in English language; (6) human studies.

### 2.3 Data extraction

Two independent reviewers were responsible for selecting and evaluating studies according to the aforementioned criteria. From each eligible study, the following information was extracted: authors and year, total number of cases, sample type, stage, position, methylation detection methods, methylation level (%), methylation threshold (%), length of follow-up (months), country, clinical endpoint, HR, 95 % CI and Outcome.

### 2.4 Statistical analysis

Forest plots and summary statistics of the pooled effect of HR and the corresponding 95 % CI were reported for each outcome. Cochran's Q test and heterogeneity statistic, I2, were calculated using inverse-variance and Dersimonian and Laird methods. The random effects model was then applied to studies with high heterogeneity. The possibility of publication bias was calculated using Egger's test and funnel plots. All plots and statistical results were obtained using the Stata 17.0 statistical software (StataCorp. 2021. Stata: Released 17. Statistical Software. College Station, TX: StataCorp LLC). Statistical significance was set at 0.05.

## 3 Results

### 3.1 Literature search and study selection

To assess the literature and exclude duplicates, 1111 articles retrieved by electronic searching were imported into ENDNOTE software. After removing 292 duplicate articles and 539 unrelated papers, the abstracts of 280 remaining articles were reviewed. 204 articles were eliminated due to divergence from the search question or the use of cell lines or animal models. 76 records remained eligible for full-text assessment, which led to the exclusion of an additional 57 articles, leaving 19 studies in the final analysis. The list of studies with eligible TTE analysis data and their general features are shown in Table 1, Table 2.

**Table 1:** Features of the studies included in the systematic review and meta-analysis.

No	Authors and Year	Total number of cases	Sample	Stage(s)	Tumor Position	Methylation detection methods	Methylation level (%)	Methylation threshold (%)	Length of follow-up (months)
1	Sahnane et al., 2015 [16]	220	FFPE	I-IV	Colon and rectum	PCR and pyrosequencing	54.3 (mean)	**NR**	**NR**
2	Chen et al., 2016 [12]	336	FFPE	II-III	Colon and rectum	PCR and pyrosequencing	52.64 (median) Range 29.81–8.73	54.62	50.30 (mean)
3	Ye-Young Rhee, 2012 [13]	207	FFPE	I-IV	Colon and rectum	PCR and pyrosequencing	58.13 (mean) Range 28.79 -70.67	53	46 (mean)
4	Shuji Ogino, 2008 [14]	643	FFPE	I-IV	Colon and rectum	PCR and pyrosequencing	61.2 (mean)	75	60
5	Joong Bae Ahn, 2011 [17]	161	Tissue	III	Proximal colon	PCR and pyrosequencing	50.1	**NR**	46
6	Marina Antelo, 2012 [18]	343	FFPE	I-IV	Colon and rectum	PCR and pyrosequencing	59.97	65	39
7	Takafumi Naito, 2014 [19]	1386	FFPE	I-IV	Colon and rectum	PCR and pyrosequencing	**NR**	50.7	39.6
8	Tai-Chuan Kuan, 2018 [9]	143	Blood	I-IV	Colon and rectum	PCR and pyrosequencing	60.5	60	61.2
9	Hatim Boughanem, 2020 [20]	67	FFPE	I-IV	Colon and rectum	PCR and pyrosequencing	**NR**	52.41	80
10	Changhua Zhuo, 2010 [21]	127	Fresh Frozen tissue	I-IV	Colon and rectum	pyrosequencing	64.62	64.47	65
11	Yun-Ting Lou, 2014 [22]	129	FFPE	III	Colon and rectum	PCR and pyrosequencing	63.3 (mean)	70.15	24
12	Kazuyuki Kawakami, 2010 [23]	155	FFPE	II-III	Colon and rectum	methylation-specific real-time PCR	84.3	84.3	58
13	A Murata, 2013 [24]	134	FFPE	I-IV	Colon and rectum	PCR and pyrosequencing	67.3	69	46.8
14	Mami Kaneko, 2016 [25]	40	FFPE	I-IV	Colon and rectum	PCR and pyrosequencing	47.4	51.7	**NR**
15	Kosuke Mima, 2016 [26]	1317	FFPE	I-IV	Colon and rectum	PCR and pyrosequencing	63.4	65	144
16	Kentaro Inamura, 2014 [27]	1211	FFPE	I-IV	Colon and rectum	PCR and pyrosequencing	62.7	**NR**	151
17	Marloes Swets, 2016 [28]	164	FFPE	II	Colon	qPCR	40.7	**NR**	**NR**
18	Naohiko Akimoto, 2021 [29]	1356	FFPE	I-IV	Colon and rectum	PCR and pyrosequencing	63.6	**NR**	117 (Median)
19	A Benard, 2013 [30]	30	FFPE	I-III	Rectum	qPCR	57.4	**NR**	84

NR= Not Reported.

**Table 2:** Main outcomes of the included studies.

No	Authors and Year	Country	Clinical endpoint(s)	Outcome
1	Sahnane et al., 2015	Italy	OS	LINE-1 hypomethylation was positively associated with a poor prognosis.
2	Chen et al., 2016	Korea	RFS	Low LINE-1 methylation level (<54.62 %) was closely associated with a shorter RFS time.
3	Ye-Young Rhee, 2012	Korea	OS	Low LINE-1 methylation level were significantly associated with shorter OS time.
4	Shuji Ogino, 2008	USA	OS and CSS	LINE-1 hypomethylation was linearly associated with a statistically significant increase in colon cancer–specific mortality. Genome-wide DNA hypomethylation as measured in LINE-1 is independently associated with poor survival among patients with colon cancer.
5	Joong Bae Ahn, 2011	Korea	DFS	Low methylation of LINE-1 in the proximal colon cancer was associated with a trend towards shorter DFS.
6	Marina Antelo, 2012	USA	OS	Comparison to patients with< 65 % LINE-1 methylation, those with >65 % LINE-1 methylation had significantly better OS.
7	Takafumi Naito, 2014	Japan	CSS	Significantly higher mortality was observed in patients LINE-1 hypomethylation compared with those with LINE-1 hypermethylation. LINE-1 hypomethylation (Q4 cases) was significantly associated with unfavorable CSS.
8	Tai-Chuan Kuan, 2018	Taipei	DFS and OS	DFS and OS of patients with LINE-1 hypomethylation tumors were significantly lower than those of patients with normal LINE-1 methylation tumors.
9	Hatim Boughanem, 2020	Spain	DFS and OS	The low LINE-1 methylation group had a worse OS and DFS rate than the high LINE-1 methylation group. LINE-1 methylation was hypermethylated in treated patients in comparison with non-treated patients.
10	Changhua Zhuo, 2015	China	CCS	Patients with a lower LMR (LINE-1 methylation Rate) had a significantly worse survival rate among certain subgroups of patients with colon cancer.
11	Yun-Ting Lou, 2012	Taiwan	OS	Patients in the low methylation group had shorter period of DFS.
12	Kazuyuki Kawakami, 2010	Japan	OS	High LINE-1 methylation was associated with a trend for longer survival in the overall patient group.
13	A Murata, 2013	Japan	OS	In liver metastatic CRC the hypomethylation group experienced an overall mortality similar to that of the hypermethylation group.
14	Mami Kaneko, 2016	Japan	PFS, OS and OPS	Patients with LINE-1 hypomethylation showed significantly worse PFS and OS following chemotherapy compared to patients with high methylation.
15	Kosuke Mima, 2016	USA	OS, CSS	tumor LINE-1 hypomethylation was associated with higher colorectal cancer-specific mortality in proximal colon cancer, but not in distal colon cancer. Tumor LINE-1 hypomethylation was associated with higher overall mortality in proximal colon cancer.
16	Kentaro Inamura, 2014	USA	OS, CSS	LINE-1 hypomethylation was associated with higher CRC-specific mortality.
17	Marloes Swets, 2016	Netherlands	OS, DFS, RFS	Patients with LINE-1 hypomethylated tumors had a significantly worse OS compared to patients with a higher level of LINE-1 tumor DNA methylation. No significant effect on RFS was observed.
18	Naohiko Akimoto, 2021	USA	OS, CSS	The tumor LINE-1 methylation level in metastatic tumors appeared to be lower than that in primary tumors in CRC.
19	A Benard, 2013	Netherlands	OS, RFS	Patients with low-LINE-1 methylation have shorter survival times and a higher chance of tumor recurrence than patients with a higher methylation percentage. Significant differences were observed for OS, overall recurrence and distant RFS.

### 3.2 Characteristics of included studies

**Fig. 1:**
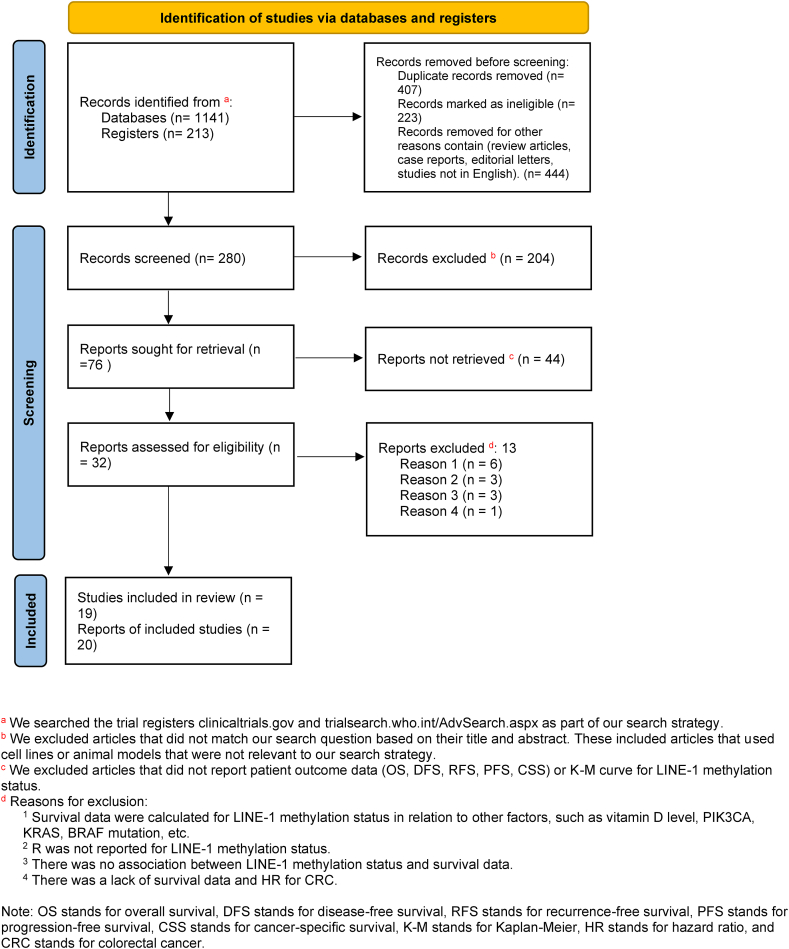
Flow diagram of literature search and study selection according to PRISMA 2020.

Nineteen studies were selected for participating in this systematic review and meta-analysis, following a rigorous selection process that conformed to the PRISMA 2020 guidelines, as illustrated in Figure_1. These studies, published between 2008 and 2021, contain information about the percentage of LINE1 methylation in 8169 CRC patients across various stages and geographical locations. This diverse cohort included both CRC patients and control subjects, representing a broad array of geographical locales. Among the included studies in 13 articles, the tumor located in colon and rectum, in 2 studies in colon and in one located at the rectum. A variety of sample types were subjected to DNA extraction, including fresh frozen tissue, blood, and formalin-fixed paraffin-embedded (FFPE) specimens. Regarding the quantification methods of LINE-1 methylation 16 studies used polymerase chain reaction (PCR) and pyrosequencing, two papers used quantitative polymerase chain reaction (qPCR) and one used methylation-specific real-time PCR and reported methylation levels in percentage terms. Notably, the studies exhibited heterogeneity in their methylation level reporting, with some delineating cut-off values while others detailed low and high ranges. The LINE-1 methylation levels reported spanned a range, with mean or median values oscillating between 40.2 % and 84.3 %. According to the strategies of each study, patients were followed for a specified period, and the relationship between the LINE-1 methylation level and one or more of the surrogate endpoints, such as OS, CSS, DFS and RFS were evaluated and reported as HR. The pivotal characteristics of the studies are concisely summarized in Table 1.

Correlation between LINE-1 Methylation and Survival Outcomes.

### 3.3 Overall survival

**Fig. 2:**
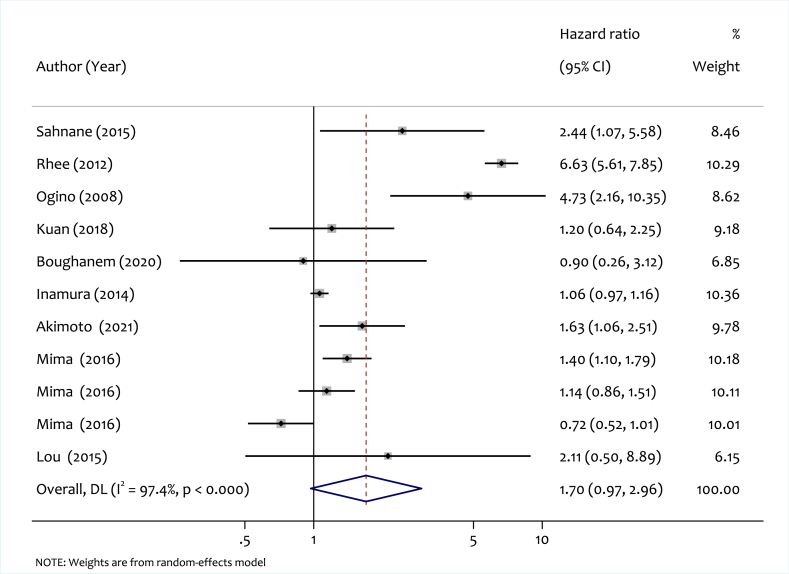
Forest plot for pooled HR and the corresponding 95 % CI of LINE-1 hypomethylation for OS among CRC patients.

A meta-analysis of 11 studies was conducted to explore the relationship between LINE-1 methylation level and OS in CRC patients. The forest plot, depicted in Figure_2, presents both study-specific and pooled HR with 95 % CIs comparing low versus high LINE-1 methylation. The pooled HR was 1.7 (95 % CI: 0.97–2.96, p < 0.001), indicating no significant association between LINE-1 methylation level and OS, alongside substantial heterogeneity (I^2^ = 97.4 %, p < 0.000).

**Fig. 3:**
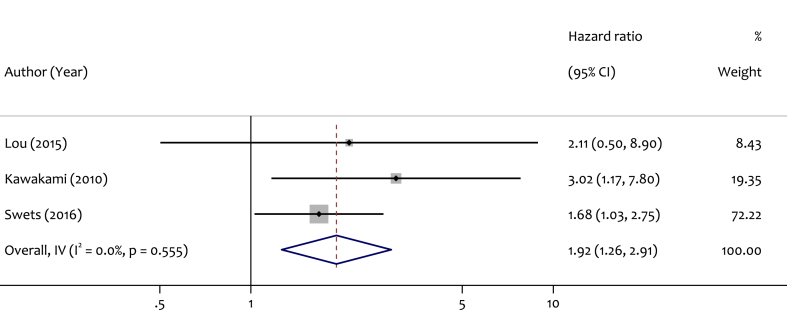
Forest plot for pooled HR and the corresponding 95 % CI of LINE-1 hypomethylation for OS among stage II and III CRC patients.

When the analysis was restricted to patients with stage II and III CRC, 3 studies were included. The pooled HR was 1.92 (95 % CI: 1.26–2.91), suggesting a significant association between lower LINE-1 methylation and poorer OS in this patient subgroup. No heterogeneity was observed among these studies (I^2^ = 0.0 %, p = 0.55). The information related to this data is shown in Figure_3.

### 3.4 Disease-free survival

**Fig. 4:**
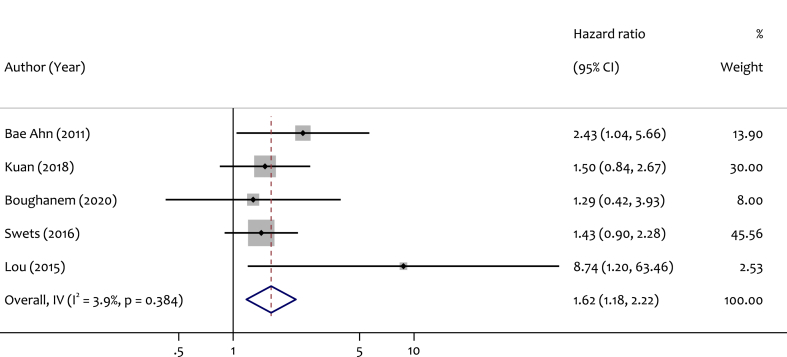
Forest Plot showing pooled HR and corresponding 95 % CI for LINE-1 Hypomethylation and DFS endpoint among CRC patients.

In analyzing the relationship between LINE-1 methylation level and DFS, 5 studies were included in the meta-analysis. The pooled HR was 1.62 (95 % CI: 1.18–2.22), demonstrating a significant association between LINE-1 methylation level and DFS with minimal heterogeneity (I^2^ = 3.9 %, p = 0.38). The corresponding forest plot is shown in Figure_4.

**Fig. 5:**
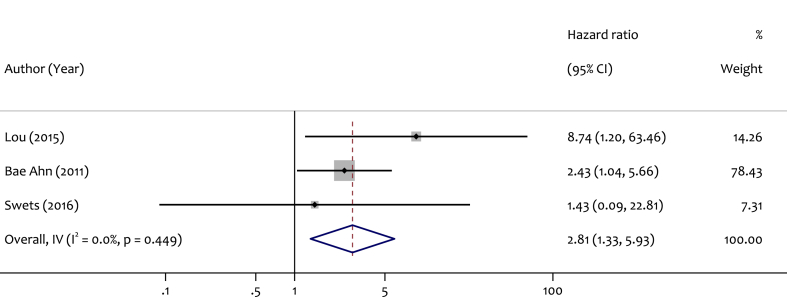
Forest plot for pooled HR and the corresponding 95 % CI of LINE-1 hypomethylation for DFS among stage II and III CRC patients.

When the analysis was restricted to patients with stage II and III CRC, 3 studies were included. The pooled HR was 2.81 (95 % CI: 1.33–5.93), suggesting a significant association between lower LINE-1 methylation and poorer DFS in this patient subgroup. No heterogeneity among studies was found (I^2^ = 0.0 %, p = 0.44). The information related to this data is shown in Figure_5.

### 3.5 Cancer-specific survival

**Fig. 6:**
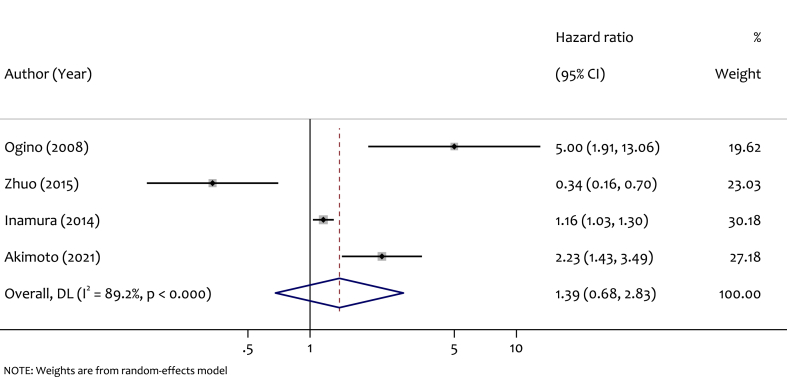
Forest plot for pooled HR and the corresponding 95 % CI of LINE-1 hypomethylation for CCS among CRC patients.

The meta-analysis included 4 studies to investigate the correlation between LINE-1 methylation level and CSS in CRC. As shown in Figure_6, the forest plot for study-specific and pooled HRs and 95 % CIs indicated a pooled HR of 1.39 (95 % CI: 0.68–2.83), with no significant association found between LINE-1 methylation level and CSS. A high level of heterogeneity was existing among the studies (I^2^ = 89.2 %, p < 0.000).

### 3.6 Assessment of publication bias

**Fig. 7:**
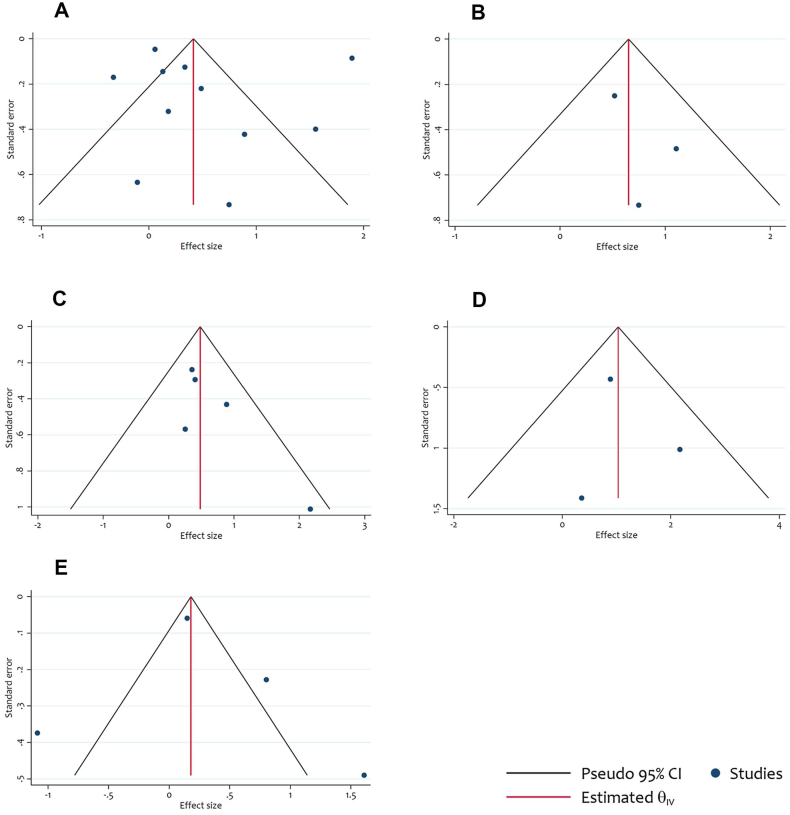
Funnel plots for assessment of publication bias in the meta-analysis of the association between LINE-1 hypomethylation and survival endpoints in CRC patients. (A) publication bias for OS (B) publication bias for OS among stage II and/or III (C) publication bias for DFS (D) publication bias for DFS among stage II and/or III (E) publication bias for CSS.

To evaluate potential publication bias, we conducted Egger's linear regression and funnel plot analyses. The results did not reveal any statistically significant publication bias for the OS (p = 0.88), stage II and/or III OS (p = 0.44), DFS (p = 0.12), stage II and/or III DFS (p = 0.89), and CSS (p = 0.74) endpoints. The funnel plots are presented in Figure_7.

## 4 Discussion

A significant portion, around 10 %, of all new cancer cases and cancer deaths each year globally are caused by CRC [31]. The frequency of CRC is expected to keep rising, particularly in less developed areas of the world. This anticipated increase is attributable to changing population demographics and aging [32]. Therefore, in order to develop more personalized strategies for CRC prevention, the screening tests must attain high accuracy by employing novel prognostic and predictive biomarkers [20]. CRC is a heterogeneous and complex disease characterized by the accumulation of genetic and epigenetic alterations over 10–20 years, leading to the alteration of normal glandular epithelium into early and advanced cancer [4,33]. The general agreement is that in CRC, epigenetic changes happen earlier and more frequently than genetic changes [34]. Epigenetics is defined as heritable variations in gene expression that do not lead to permanent changes in the DNA sequence and is crucial during embryonic development, imprinting, and tissue differentiation. Epigenetic modifications, such as DNA methylation, post-translational histone modifications, and microRNAs post-transcriptional regulation, play a key role in CRC development. disruption of epigenetic mechanisms can change cellular homeostasis and promote the growth of tumors [4,35]. A common observation in various cancer types, including CRC is global DNA hypomethylation which plays a key role in the beginning and progression of cancer. One potential mechanism by which DNA hypomethylation contributes to cancer development involves the activation of LINE-1 retrotransposons and consequent genomic instability. LINE-1 elements have the capacity to insert copies of themselves throughout the human genome, generating new genetic sequences that drive human evolution [36]. LINE-1 elements have the potential to disrupt gene function through insertional mutagenesis or aberrant splicing events when they transpose to new genomic locations. Additionally, the integration of LINE-1 sequences could lead to deletions of DNA at the target site, contributing to disease development. In normal tissues, LINE-1 elements are highly methylated and transcriptionally silenced, although a subset retains the ability to retrotranspose themselves to new integration sites. However, in cancer tissues, LINE-1 methylation levels are reduced. This hypomethylation of LINE-1 promoters has been associated with increased retrotransposition activity, induced genomic instability, and further progression of cancer. Given the high copy number and dispersed genomic distribution of LINE-1 sequences, their methylation status can serve as a surrogate marker for estimating global DNA methylation levels. The effects of LINE-1 methylation level on CRC progression and patient survival have been investigated in various studies [9].

Our study advances Ye et al. (2017)'s meta-analysis on LINE-1 methylation in CRC, addressing previous limitations in methylation classification and survival analysis. We included 8169 patients from 19 studies, revealing no significant impact on OS in the general CRC cohort. However, stage II/III patients showed poorer outcomes with LINE-1 hypomethylation, highlighting its prognostic value in advanced CRC. This update provides a refined prognostic tool, contributing to personalized CRC patient care [37]. The present systematic review and meta-analysis aimed to comprehensively evaluate the prognostic significance of LINE-1 methylation level in CRC patients. By synthesizing data from 19 eligible studies encompassing a diverse cohort of CRC patients from various geographical regions, we sought to elucidate the relationship between LINE-1 hypomethylation and crucial survival outcomes, namely OS, DFS, and CSS.

Our meta-analysis revealed no statistically significant association between LINE-1 hypomethylation and OS in the overall CRC patient population. However, when the analysis was restricted to stage II and III CRC patients, a significant correlation emerged, with LINE-1 hypomethylation conferring an increased risk of poorer OS. This finding aligns with previous reports suggesting that LINE-1 hypomethylation is associated with more aggressive tumor behavior and a higher likelihood of metastasis, potentially contributing to reduced survival rates in these advanced stages [14,27].

Furthermore, our analysis demonstrated a significant association between LINE-1 hypomethylation and inferior DFS in the overall CRC population, as well as in the subgroup of stage II and III patients. These findings corroborate the notion that LINE-1 hypomethylation may promote tumor progression and increase the risk of disease recurrence, potentially through mechanisms such as genomic instability and aberrant transcriptional activation of oncogenes [6,10].

Interestingly, our analysis did not reveal a significant association between LINE-1 hypomethylation and CSS in CRC patients. This observation suggests that while LINE-1 hypomethylation may contribute to disease progression and recurrence, its impact on cancer-specific mortality may be influenced by other factors, such as treatment response, comorbidities, and overall health status.

It is noteworthy that substantial heterogeneity was observed among the included studies, particularly in the analyses of OS and CSS. This heterogeneity may be attributed to variations in study populations, sample types (e.g., fresh frozen tissue, FFPE), methylation detection methods, and cut-off values for defining LINE-1 hypomethylation. Furthermore, differences in treatment regimens, follow-up durations, and the inclusion of patients with different tumor stages and locations (e.g., proximal vs. distal colon cancer) might have contributed to the observed heterogeneity.

Despite the comprehensive nature of our analysis, we acknowledge several restrictions that should be considered when evaluating our findings. The limitations of our meta-analysis include variability in methylation measurement methods, as different studies utilized techniques such as PCR, pyrosequencing, and methylation-specific real-time PCR, leading to inconsistencies in reported LINE-1 methylation levels and complicating comparisons. Additionally, there is a lack of standardized cut-off points for distinguishing between hypomethylation and hypermethylation of LINE-1, which may affect patient classification and the interpretation of survival outcomes. Significant heterogeneity among the included studies regarding patient demographics, tumor stages, treatment regimens, and follow-up durations may further impact the generalizability of our findings. Furthermore, despite a thorough literature search, the potential for publication bias exists, as studies with negative or inconclusive results may be underrepresented. Our analysis also primarily focused on LINE-1 methylation levels, while other important prognostic factors, such as genetic mutations, treatment response, and comorbidities, were not consistently reported, hindering a comprehensive understanding of factors influencing survival outcomes in CRC patients. Lastly, many studies included were retrospective in nature, which can introduce biases related to patient selection and data collection, potentially affecting the reliability of our conclusions.

Our initial manuscript did not address the end-point of time to recurrence (TTR) due to limited data. The significance of TTR, as outlined by DATECAN, includes anastomotic and metastatic relapses, death with evidence of recurrence, and death from colon cancer. Similarly, for RFS, which encompasses all causes of death and relapses, there was insufficient data for analysis.

The heterogeneity in LINE-1 methylation levels reported in various studies makes establishing a definitive cut-off point a complex task. The range of LINE-1 methylation spans from 22 % to 88 %, with most studies reporting mean or median values between 47.4 % and 67.3 %. Typically, methylation levels below 50–54 % are classified as low, while levels above 60–70 % are considered high, and those within 55–65 % fall into an intermediate category. Our systematic review and meta-analysis suggest that a 50 % cut-off does not adequately represent severe hypomethylation. A threshold between 60 and 70 % aligns better with the median methylation levels observed in healthier samples. Confirmatory research is essential to determine the most accurate cut-off for LINE-1 methylation in CRC diagnosis and prognosis.

Despite the limitations, our study provides a comprehensive synthesis of the available evidence, highlighting the potential prognostic utility of LINE-1 methylation status in CRC. The findings suggest that LINE-1 hypomethylation may serve as a valuable biomarker for predicting disease recurrence and survival outcomes, particularly in stage II and III CRC patients. Incorporating LINE-1 methylation analysis into clinical decision-making could aid in risk stratification and personalized treatment strategies.

Future studies should aim to establish standardized cut-off values and methodologies for LINE-1 methylation assessment, facilitating cross-study comparisons and meta-analyses. Additionally, prospective studies investigating the prognostic value of LINE-1 methylation in conjunction with other molecular and clinicopathological features are warranted to gain a comprehensive understanding of its role in CRC prognosis and treatment response.

## 5 Conclusion

This systematic review and meta-analysis provide insights into the prognostic significance of LINE-1 hypomethylation in CRC, highlighting its association with poorer survival outcomes, particularly in advanced stages. While further research is needed to address the limitations and heterogeneity observed, our findings support the potential utility of LINE-1 methylation level as a prognostic biomarker in CRC management.

## CRediT authorship contribution statement

**Akbar Bagheri:** Writing – review & editing, Writing – original draft, Project administration, Methodology, Investigation, Conceptualization. **Tohid Jafari-Koshki:** Writing – review & editing, Software, Methodology, Investigation, Formal analysis, Data curation, Conceptualization. **Leila Alizadeh:** Writing – review & editing, Investigation, Conceptualization. **Mortaza Raeisi:** Writing – review & editing, Investigation. **Yaghoub Moaddab:** Investigation. **Abbas Karimi:** Writing – review & editing, Writing – original draft, Supervision, Methodology, Funding acquisition, Data curation, Conceptualization.

## Ethics statement

This systematic review and meta-analysis utilized published data from indexed journals, and therefore does not involve direct interaction with human participants. The analysis was conducted under ethical approval IR.TBZMED.REC.1402.164. All included studies adhered to ethical standards, including informed consent from participants where applicable, and ethical approval from relevant institutional review boards.

## Data availability statement

The dataset used and analyzed during this study is available from the corresponding author upon reasonable request.

## Funding

This work is part of the Ph.D. project supported by the Tabriz University of Medical Sciences under grant number 71130.

## Declaration of competing interest

The authors declare that they have no known competing financial interests or personal relationships that could have appeared to influence the work reported in this paper.
