# Supplementary material for: LINE-1 methylation status and survival outcomes in colorectal cancer patients: A systematic review and meta-analysis
Source: Heliyon. 2025 Jan 31;11(3):e42410. doi: 10.1016/j.heliyon.2025.e42410 (PMC11849065; doi:10.1016/j.heliyon.2025.e42410)
Supplement: Multimedia component 1 [file mmc1.docx]

**Supplementary Table 1:** search strategy and terms used for the systematic literature search on the association between LINE-1 methylation and survival outcomes in colorectal cancer

| **Words** | **Mesh Word** | **Entry** |
| --- | --- | --- |
| Colorectal Cancer | Colorectal Neoplasms | Colorectal Neoplasm  Neoplasm, Colorectal  Neoplasms, Colorectal  Colorectal Tumors  Colorectal Tumor  Tumor, Colorectal  Tumors, Colorectal  Colorectal Cancer  Cancer, Colorectal  Cancers, Colorectal  Colorectal Cancers  Colorectal Carcinoma  Carcinoma, Colorectal  Carcinomas, Colorectal  Colorectal Carcinomas |
| LINE 1 | Long Interspersed Nucleotide Elements | LINE Repeat Sequences  LINE Repeat Sequence  Repeat Sequence, LINE  Repeat Sequences, LINE  Sequence, LINE Repeat  Sequences, LINE Repeat  Long Interspersed DNA Sequence Elements  LINE-1 Elements  Element, LINE-1  Elements, LINE-1  LINE 1 Elements  LINE-1 Element  L1 Elements  Element, L1  Elements, L1  L1 Element  Jockey Elements  Element, Jockey  Elements, Jockey  Jockey Element |
| Survival Analysis | Survival Analysis | Analysis, Survival  Analyses, Survival  Survival Analyses |
| Disease-Free Survival | Disease-Free Survival | Disease Free Survival  Survival, Disease-Free  Survival, Disease Free |
| Progression-Free Survival | Progression-Free Survival | Progression Free Survival  Survival, Progression-Free  Event-Free Survival  Event Free Survival  Survival, Event-Free |
| Recurrence | Recurrence | Recurrences  Relapse  Relapses  Recrudescence  Recrudescences |
| Kaplan-Meier Estimate | Kaplan-Meier Estimate | Estimate, Kaplan-Meier  Kaplan-Meier Analysis  Analysis, Kaplan-Meier  Kaplan Meier Analysis  Kaplan-Meier Test  Kaplan Meier Test  Test, Kaplan-Meier  Product-Limit Method  Method, Product-Limit  Methods, Product-Limit  Product Limit Method  Product-Limit Methods  Kaplan-Meier Survival Curves  Kaplan Meier Survival Curves  Kaplan-Meier Survival Curve  Curve, Kaplan-Meier Survival  Curves, Kaplan-Meier Survival  Kaplan Meier Survival Curve  Survival Curve, Kaplan-Meier  Survival Curves, Kaplan-Meier |
| Prognosis | Prognosis | Prognoses  Prognostic Factors  Prognostic Factor  Factor, Prognostic  Factors, Prognostic |
