# Supplementary material for: LINE-1 methylation status and survival outcomes in colorectal cancer patients: A systematic review and meta-analysis
Source: Heliyon. 2025 Jan 31;11(3):e42410. doi: 10.1016/j.heliyon.2025.e42410 (PMC11849065; doi:10.1016/j.heliyon.2025.e42410)
Supplement: Multimedia component 2 [file mmc2.docx]

**Supplementary Table 2:** summary of the search results across different databases on the association between LINE-1 methylation and survival outcomes in colorectal cancer

| **Keywords** | **Number of results** | **Database** |
| --- | --- | --- |
| (“Long Interspersed Nucleotide Elements" [MeSH Terms] OR (“Long Interspersed Nucleotide Elements" [All Fields] OR “LINE 1" [All Fields])) AND (“colorectal neoplasms" [MeSH Terms] OR (“colorectal" [All Fields] AND “neoplasms" [All Fields]) OR “colorectal neoplasms" [All Fields] OR (“colorectal" [All Fields] AND “cancer" [All Fields]) OR “colorectal cancer" [All Fields] OR (“colorectal neoplasms" [MeSH Terms] OR (“colorectal" [All Fields] AND “neoplasms" [All Fields]) OR “colorectal neoplasms" [All Fields] OR (“colorectal" [All Fields] AND “carcinoma" [All Fields]) OR “colorectal carcinoma" [All Fields])) | 241 | PubMed |
| (“Long Interspersed Nucleotide Elements" [MeSH Terms] OR (“Long Interspersed Nucleotide Elements" [All Fields] OR “LINE 1" [All Fields])) AND (“colorectal neoplasms" [MeSH Terms] OR (“colorectal" [All Fields] AND “neoplasms" [All Fields]) OR “colorectal neoplasms" [All Fields] OR (“colorectal" [All Fields] AND “cancer" [All Fields]) OR “colorectal cancer" [All Fields] OR (“colorectal neoplasms" [MeSH Terms] OR (“colorectal" [All Fields] AND “neoplasms" [All Fields]) OR “colorectal neoplasms" [All Fields] OR (“colorectal" [All Fields] AND “carcinoma" [All Fields]) OR “colorectal carcinoma" [All Fields])) AND (“Survival Analysis"[MeSH Terms] OR (“Survival Analysis" [All Fields] OR “overall survival" [All Fields] OR “Disease-Free Survival" [MeSH Terms] OR “Disease-Free Survival" [All Fields] OR “Progression-Free Survival" [MeSH Terms] OR “Progression-Free Survival" [All Fields] OR “Kaplan-Meier Estimate" [MeSH Terms] OR “Kaplan-Meier Estimate" [All Fields] OR “time to recurrence" [All Fields] OR “cancer-specific survival" [All Fields] OR “prognosis" [MeSH Terms] OR “prognosis" [All Fields] OR “prognoses" [All Fields] OR “outcome" [All Fields] OR “outcomes" [All Fields] OR “Prognostic Value" [All Fields] OR “Prognostic Factor" [All Fields] OR “Prognostic Indicator" [All Fields] OR “Recurrence Risk" [All Fields] OR “Predictive for Outcome"[All Fields] OR “recurrence"[All Fields])) | 115 | PubMed |
| (“Colorectal Neoplasms” OR “Colorectal cancer” OR “CRC” ) AND (“Long Interspersed Nucleotide Elements” OR “LINE-1”) | 770 | Science Direct |
| Long Interspersed Nucleotide Elements OR LINE-1 AND Colorectal cancer OR colorectal carcinoma | 15 | cochranelibrary |
| Colorectal Neoplasms AND LINE-1 | 188 | <https://clinicaltrials.gov/> |
| Colorectal Neoplasms AND LINE-1 | 25 | [https://trialsearch.who.int](https://trialsearch.who.int/) |
| All | 1354 |  |
